# Anaerobic Digestion of *Laminaria japonica* Waste from Industrial Production Residues in Laboratory- and Pilot-Scale

**DOI:** 10.3390/md13095947

**Published:** 2015-09-18

**Authors:** Yann Nicolas Barbot, Claudia Thomsen, Laurenz Thomsen, Roland Benz

**Affiliations:** 1 Department of Life Sciences and Chemistry, Jacobs University Bremen, Campus Ring 1, Bremen 28759, Germany; E-Mail: r.benz@jacobs-university.de; 2 Phytolutions GmbH, Campus Ring 1, Bremen 28759, Germany; E-Mail: c.thomsen@phytolutions.com; 3 Department of Physics and Earth Sciences, Jacobs University Bremen, Campus Ring 1, Bremen 28759, Germany; E-Mail: l.thomsen@jacobs-university.de

**Keywords:** macroalgae, waste management, biogas, *Laminaria japonica*, industrial residuals, biomethane, flue gas condensate, maize co-digestion, thermo-acidic pretreatment

## Abstract

The cultivation of macroalgae to supply the biofuel, pharmaceutical or food industries generates a considerable amount of organic residue, which represents a potential substrate for biomethanation. Its use optimizes the total resource exploitation by the simultaneous disposal of waste biomaterials. In this study, we explored the biochemical methane potential (BMP) and biomethane recovery of industrial *Laminaria japonica* waste (LJW) in batch, continuous laboratory and pilot-scale trials. Thermo-acidic pretreatment with industry-grade HCl or industrial flue gas condensate (FGC), as well as a co-digestion approach with maize silage (MS) did not improve the biomethane recovery. BMPs between 172 mL and 214 mL g^−1^ volatile solids (VS) were recorded. We proved the feasibility of long-term continuous anaerobic digestion with LJW as sole feedstock showing a steady biomethane production rate of 173 mL g^−1^ VS. The quality of fermentation residue was sufficient to serve as biofertilizer, with enriched amounts of potassium, sulfur and iron. We further demonstrated the upscaling feasibility of the process in a pilot-scale system where a CH_4_ recovery of 189 L kg^−1^ VS was achieved and a biogas composition of 55% CH_4_ and 38% CO_2_ was recorded.

## 1. Introduction

### 1.1. Marine Macroalgae for Biogas Production

Dwindling reserves of fossil combustibles, the strong dependency of global economies on these fuels and the global increase in energy demand has urged governments around the world to rethink the concept of providing long-term energy security [1]. Energy from renewable sources, such as biofuels, represents a possible alternative to guarantee a secured energy supply in the future and would allow depleted resources to be replenished accordingly [2]. Anaerobic digestion is a microbial conversion process in which organic matter is transformed into gaseous biomethane and carbon dioxide. Currently, biogas plants are largely operated on terrestrial energy crops such as maize silage [3]. The increase in biomass demand, relative to the increasing number of operating plants [4], has led to the intense cultivation of energy crops. This, in turn, provokes a variety of environmental, ethical and social problems, such as the food-fuel competition debate [5], the increased need for fresh water and fertilizer [6,7] and enhanced greenhouse gas emissions [6,8]. These conflicts lower the sustainable nature and the acceptance of biofuels within the society. Beringer *et al*. [9] point out that this increasing demand for food and bio-energy production for the rising world population will deepen the conflict, since the currently available land will develop a strong yield reduction due to climate change. Using marine biomass, as opposed to terrestrial crops, would avoid many of the above mentioned friction points by simply using the enormous biomass growth potential of the oceans where approximately 50% of the annual global biomass grows [10] and in which only 2% contributes to the global food supply [11]—in particular, macroalgae exhibit a number of promising features, such as high growth rates [12], high production yields [13], growth in salt water, low lignocellulose content [14] and high soluble carbohydrate composition [15,16]. These properties make them an interesting candidate for biomethane production. Nowadays, seaweeds are the subject of keen interest in many industrial sectors, with promising trends towards increasing global production [17,18]. The utilization and manufacturing of seaweeds in comestible, pharmaceutical, and cosmetic industries generates considerable waste streams of organic residues which need to be disposed. A convenient solution to manage the waste would be its application as a substrate in biomethane production. The technology is suitable for waste management [19,20] and the conversion process can be perfectly operated with low quality or residual biomass [21,22].

### 1.2. Anaerobic Digestion of Seaweeds

Macroalgae exhibit an interesting theoretical biomethane potential due to their particular chemical composition [23]. They consist of a large carbohydrate fraction (up to 50%–60%), a varying mineral (7%–38%) and protein (10%–47%) composition and feature a low lipid content (1%–3%) [15,16]. However, the chemical composition of seaweeds shows fluctuating variations across species [24] and is dependent on the season of the year [24,25], thereby strongly affecting biogas quantity and quality [26,27]. The outstanding potential for anaerobic digestion (AD) [28,29] lies in their carbohydrate fraction, which is mainly composed of readily soluble and easily hydrolyzable sugars (e.g., laminarin, mannitol) [2,14,30] as well as the structural polysaccharide alginic acid [31]. This accounts in particular for brown algae, often also referred to as seaweed or kelp [14,32]. The biomethane and biohydrogen potential of brown seaweeds has recently been studied in various works [33,34,35], its quality for biomethane production shown to be just as comparable to that of terrestrial plants [36]. Brown seaweeds mostly lack lignin and contain only little quantities of cellulose [14], two compounds abundant in terrestrial biomass which are difficult to degrade for microorganisms [37]. Laboratory trials have shown good biomethane recovery from AD of seaweeds [5,14,32]. Recent works reported biomethane yields between 260 and 280 mL g^−1^ VS for AD of macrophyte biomass from the *Laminaria* genera [38]. Different pretreatment (PT) methods for biomass disruption, such as mechanical, thermal, enzymatic and thermo-chemical treatment, have been shown to successfully improve biomethane recovery with average values between +19% and +68%, sometimes even up to +140% [22,33,39]. Additionally, co-digestion trials of seaweeds together with cattle manure, wheat straw or dairy slurry improved the overall quality of biomass conversion [23,33,34]. The potential for utilizing industrial seaweed wastes for the production of methane has scarcely been tested, but a few trials have shown promising results. Edyvean *et al*. [40] yielded 320 mL·g^−1^ VS biogas with 62% methane from seaweed waste following alginate extraction. Anaerobic digestion of waste sludge obtained from alginate extraction from *Laminaria hyperborea* and *Ascophyllum nodosum* yielded biomethane between 100 and 150 mL·g^−1^ VS and 70 to 280 mL·g^−1^ VS, respectively [41]. Most of the other published works related to biomethanation of macroalgae have tested the BMP in small-scale batch trials, while only a few carried out small-scale experiments in continuous mode. Proper experimentation in continuous laboratory- or pilot-scale applications in relation to long-term digestion and upscaling feasibility is scarce and was only conducted by very few authors using whole-plant beached macroalgae [35,42].

### 1.3. Marine Biomass from Industrial Waste Streams

Historically, macroalgae are typical foodstuff in Asian countries with long coastlines [32]. The technology for large-scale cultivation and farming already exist since seaweeds are a traditional part of the daily diet in these regions [18,32]. To date, the world’s largest macroalgae producers of aquaculture seaweeds are China, Indonesia, the Philippines, South Korea, North Korea, Japan and Malaysia, covering up 99% of the market share [18]. The major cultivated species belong to the *Undaria*, *Porphyra*, *Laminaria*, *Eucheuma* and *Gracilaria* genera, accounting for 76% of the total macroalgae quantity produced by aquaculture [5,14,18]. Most of the seaweeds produced (83%–90%) are used as foodstuff [43], but other industries also use macroalgae as a source material to produce agar (pharmaceutical laxative), carrageenan (toothpaste ingredient) or alginate (paper industry, textile printing, medical fiber) [2,43]. In 2011, the global seaweed industry generated a total of 21 million tons of biomaterial, which is an increase of 30% compared to 2008 [18]. In comparison to almost 850 million tons of estimated annual energy crop production [5], the seaweed market is smaller, although it exhibits a surprising potential for increasing production. In addition, other regions of the world have shown intensified interest in the aqua-cultivation of seaweeds, notably North America [44] and Europe [45]. Recent studies have dealt with exploring the macroalgae farming potential of Europe as a market for industrial high quality resources and biofuels, giving positive prospects for the European seaweed industry [32,36]. The global yield of *Laminaria japonica* which was used in this study has been increased to several hundred thousand tons during the last years, while in America and Europe the annual yield is less than 100 tons [46]. During both the harvesting and downstream processing of *Laminaria*, 10%–30% of the biomass remains as residual biomass and is either washed ashore or treated as low quality residue or as waste (Haizhibao Ocean Technology Co., Ltd., Weihai, China. Personal communication [47]). The potential of seaweeds for biogas production was evaluated by life cycle assessment (LCA) for Sicily, Italy [36] and for Nordic conditions (North Atlantic, North Sea) [48]. Such approaches reveal an interest for future industrial exploitation of European shores as seaweed production sites. Accordingly, the quantities of organic residues and waste materials should increase to allow facilitated access and supply of potential source material for biomethanation.

Using residual biomass from industrial processes offers the exceptional advantage of securing a continuous supply of algal biomaterial with consistent quality and composition. Most of the macroalgal waste material will undergo treatment during the harvesting or manufacturing process, which generally enhances the structural biomass disintegration. Pretreated material with readily fermentable organics will facilitate the subsequent microbial conversion process. Shilton and Guieysse [21] suggested in 2010 to focus on terrestrial marginal organics rather than using first-rate energy crops for biomethanation. Wellinger *et al*. [20] estimated the potential of all agricultural by-products and wastes used for biogas production in Germany as 26.5 tWh of electricity per year. This quantity would supply 7.5 million households (with 3 persons using 3500 kWh) per year [20]. Similarly, this concept could be applied to the management of aquaculture residuals, whereby biological waste is used in biogas plants to dispose of the spare material and to subsequently resupply the compounds to the natural system in the form of biofertilizer.

### 1.4. Aims of the Study

This study investigates the biochemical methane potential and the CH_4_ formation dynamics of anaerobically digested residuals from industrially processed *Laminaria japonica* (LJW). The idea is to create a disposal pathway for the accumulating industrial biowaste, combining resource recycling and the simultaneous production of biomethane. To improve the BMP from LJW, thermo-acidic pretreatment is applied to the biomass using industry-grade HCl, or acidic flue gas condensate from a middle-calorific power plant as a disposable waste product. Furthermore, the quality of a co-digestion approach of LJW together with maize silage is tested. The macroalgal substrate is supposed to partially reduce the current share of terrestrial energy crop ingestate and act in a first step as a co-substrate. To obtain reliable information about long-term AD of LJW, the algal substrate is first tested in laboratory-scale continuous anaerobic digestion. Subsequently the use of LJW is also tested in pilot-scale process mode to obtain complementary information about upscaling feasibility. In a last step, the fermentation residue after continuous AD of LJW is analyzed with regards to use as a fertilizer product. The accumulated digestate can be considered as an undesirable waste product or a valuable source of nutrients depending on its composition.

## 2. Results

### 2.1. Composition of Laminaria japonica Waste LJW and Theoretical Methane Potential

An elementary analysis of LJW showed that its general composition was in a comparable range to that of maize but with higher macronutrient concentrations for potassium (5-fold), magnesium (2.5-fold), calcium (3-fold) and sulfur (3-fold). Micronutrient concentrations were also higher in LJW for molybdenum (3-fold), iron (19-fold) and manganese (5-fold). The carbon concentration in LJW was about half of that of maize (Table 1). The quantity of heavy metals was identified according to the German Biowaste Act *Bioabfallverordnung* (BioAbfV) (Table 1). Literature values for maize were taken to put into context the gathered data and to obtain comparable benchmark values [49,50].

**Table 1:** Elementary analysis of LJW biomass and literature values for the elemental composition of maize.

Category	Element	Standard Method	LJW	Maize	Unit
BioAbfV	Lead (Pb)	DIN 38406-E6:1981-05	3	2	mg·kg^−1^ TS
	Cadmium (Cd)	ISO 5961-E19:1995-05	0.5	0.7	
	Chromium (Cr)	ISO 11885-E22:1997-11	14	0.5	
	Copper (Cu)	ISO 11885-E22:1997-11	5	4.5–5	
	Nickel (Ni)	ISO 11885-E22:1997-11	3	5	
	Mercury (Hg)	EN 12338-E31:1998-07	<0.04		
	Zinc (Zn)	ISO 11885-E22:1997-11	28	35–56	
Macronutrients	Phosphorous (P)	ISO 11885-E22:1997-11	2060	2200	
	Potassium (K)	ISO 11885-E22:1997-11	89,900	17,800	
	Magnesium (Mg)	ISO 11885-E22:1997-11	6800	2700	
	Calcium (Ca)	ISO 11885-E22:1997-11	14,000	4500	
	Sulfur (S)	ISO 11885-E22:1997-11	8590	2700	
	C/N ratio		10.5:1	~30:1	
	Total carbon	ISO 10694:1996-08	21	43	% TS
	Total nitrogen	ISO 1161:1997-05	20,000	14,000	mg·kg^−1^ TS
Micronutrients	Molybdenum (Mo)	ISO 11885-E22:1997-11	1	0.3	mg·kg^−1^ TS
	Iron (Fe)	ISO 11885-E22:1997-11	3440	184	
	Cobalt (Co)	ISO 11885-E22:1997-11	1.5	65	
	Selenium (Se)	DIN 38405-D23:1994-10	0.3		
	Manganese (Mn)	ISO 11885-E22:1997-11	150	29	

The composition of LJW was determined with regard to the carbohydrate, protein and lipid content in the biomass (Table 2). The LJW composition analysis was performed by the LUFA Nord-West institute according to standardized methods. To obtain a maximum benchmark for the output efficiency, the theoretical maximum BMP from LJW was calculated based on the individual methane recovery from each share of carbohydrate, protein and lipid as stated in the VDI-4630 guidelines [51] (Table 2). Composition analysis and elementary analysis were performed from the same sample probe.

**Table 2:** Composition and theoretical methane potential of LJW biomass.

Component	Share [%]	Theoretical CH_4_		Unit	Standard Method
		TS	VS		
Volatile solids	50.9				Described in Section 4.8.1.
*Carbohydrate*	39.2	145	285	mL·g^−1^	Calculated
*Fiber*	6.4	-	-		Weender method
*Protein*	11.4	52	102	mL·g^−1^	Kiejdahl method
*Lipid*	0.3	2	4	mL·g^−1^	VDLUFA Bd. III, Kap 5.1.1
Inorganic solids	49.1	-	-		Described in Section 4.8.1.
Total	100	199	391	mL·g^−1^	

### 2.2. BMP of Acid Hydrolysis Pretreated and Untreated LJW

**Figure 1:**
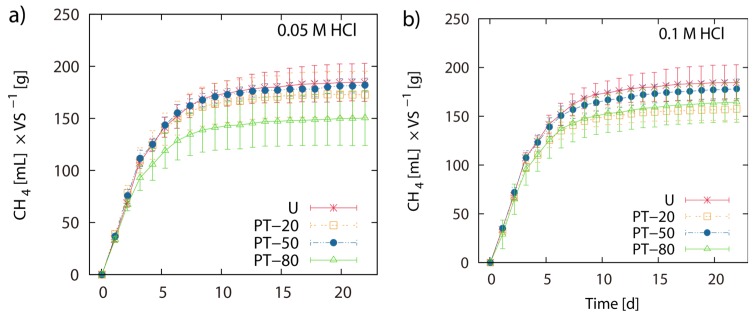

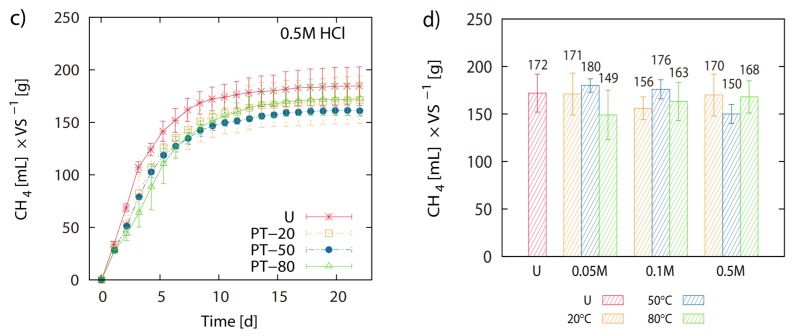
Thermo-acidic pretreatment: Net accumulated methane production of pre-treated (PT) and untreated (U) *Laminaria japonica* waste. Pre-treatment was applied for 2 h at 20 °C (PT-20), 50 °C (PT-50) and 80 °C (PT-80) in 0.05 M HCl (**a**), 0.1 M HCl (**b**) and 0.5 M HCl (**c**). (**d**) shows the histogram of the final net methane yield in mL·g^−1^ VS algae biomass. The values of the final methane yield are reported in Table 3 and were taken at Day 22.

**Figure 2:**
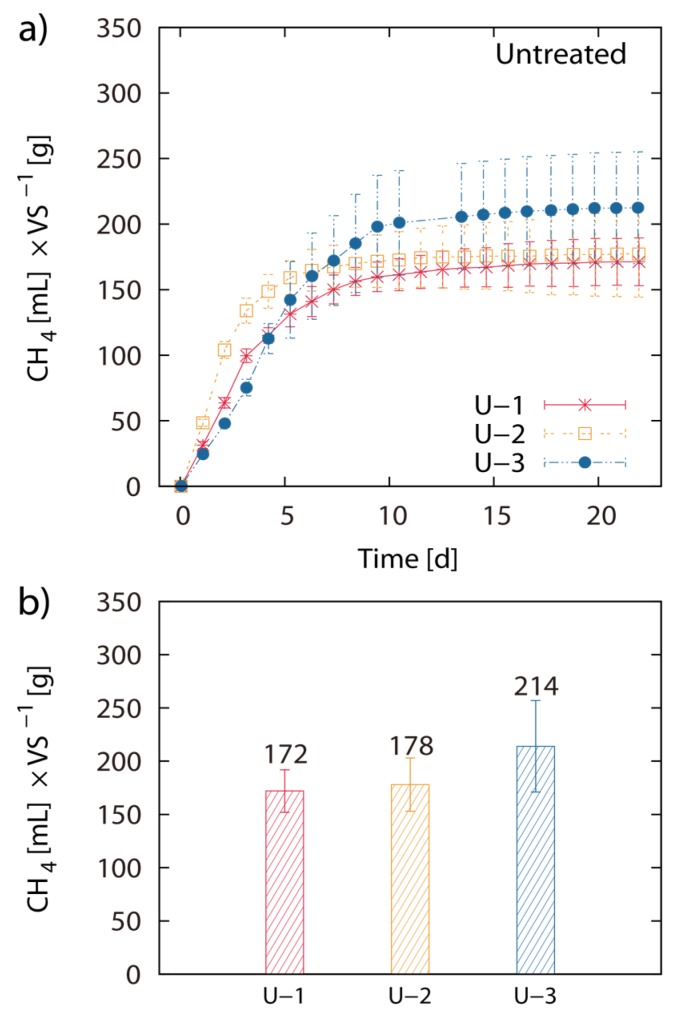
Inoculum adaptation to substrate*:* Net accumulated methane production of untreated (U) *Laminaria japonica* waste (**a**) and final net methane yield in mL per g of VS algae biomass (**b**). Results are from different succeeding trials (U-1, U-2, U-3). The values of the final methane yield are reported in Table 3 and were taken at Day 22.

**Figure 3:**
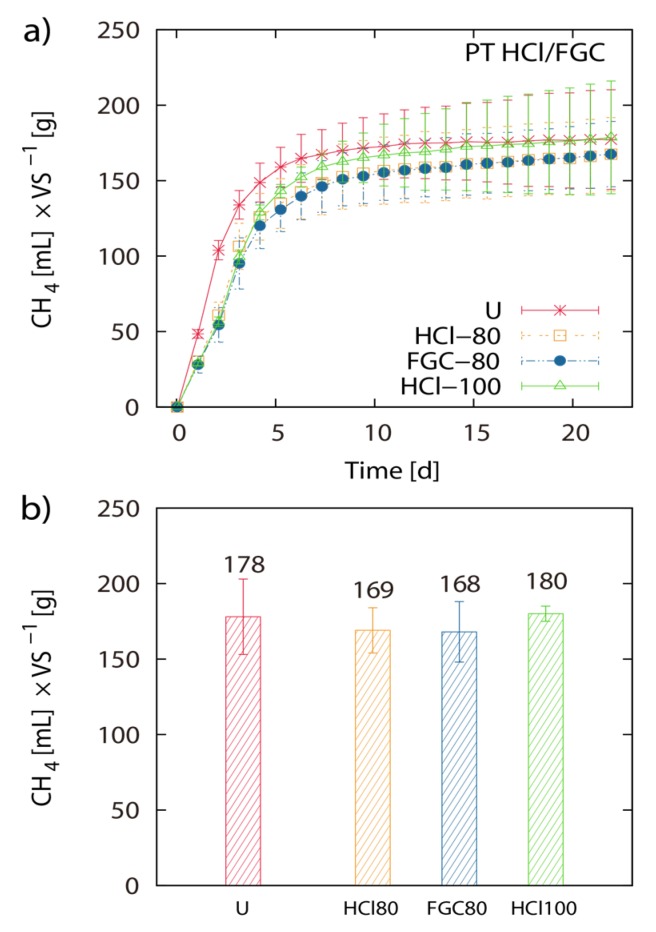
Pretreatment with flue gas condensate: Net accumulated methane production of pre-treated and untreated (U) *Laminaria japonica* waste. Pre-treatment was applied for 2 h at 80 °C in 0.2 M HCl (HCl-80) and FGC (FGC-80) and at 100 °C in HCl (HCl-100) (**a**). (**b**) shows the histogram of the final net methane yield in mL g^−1^ VS algae biomass. The values of the final methane yield are reported in Table 3 and were taken at Day 22.

Pretreatment of LJW with 0.05 M HCl at 20 °C, 50 °C and 80 °C resulted in 171 mL, 180 mL and 149 mL CH4 g^−1^ VS, respectively. Pretreatment of LJW with 0.1 M HCl at 20 °C, 50 °C and 80 °C resulted in 156 mL, 176 mL and 163 mL CH4 g^−1^ VS respectively. Pretreatment with 0.5 M HCl at 20 °C, 50 °C and 80 °C resulted in 170 mL, 150 mL and 168 mL CH4 g^−1^ VS respectively (Figure 1a–d). Total methane yield of non-pretreated U-1 (untreated LJW in experiment 1), U-2 (untreated LJW in experiment 2) and U-3 (untreated LJW in experiment 3) was 172 mL·g^−1^, 178 mL·g^−1^ and 214 mL·g^−1^ VS (Figure 2a,b). U-1, U-2 and U-3 were results from three different experiments, successive in time, where a part of the sludge from the previous experiment was reused as inoculum for the next one (U-1 for U-2; U-2 for U-3). The application of pretreatment with FGC and HCl (both with pH 1.2), at a pretreatment reaction temperature of 80 °C yielded 169 mL and 168 mL CH4 g^−1^ VS, respectively (Figure 3a,b). The results show no major differences between use of HCl or FGC for thermo-acidic pretreatment. The increase of the pretreatment reaction temperature to 100 °C (in 0.2 M HCl) showed a biomethane recovery of 180 mL g^−1^ VS. The different approaches of thermo-acidic pretreatment did not show much improvement of biomethane recovery compared to the BMP of untreated LJW. The application of some pretreatment approaches even lowered the total recovery performance. However, an increase in BMP from biomethanation of untreated LJW was observed in the successive trials when substrate-specific pre-adapted inoculum sludge was reused (Figure 3b). The BMP was increased by +3% and +24% for the U-2 and U-3 experiments in comparison with U-1.

### 2.3. BMP of LJW and Evaluation of Methane Production for Co-Digestion with Maize Silage

**Figure 4:**
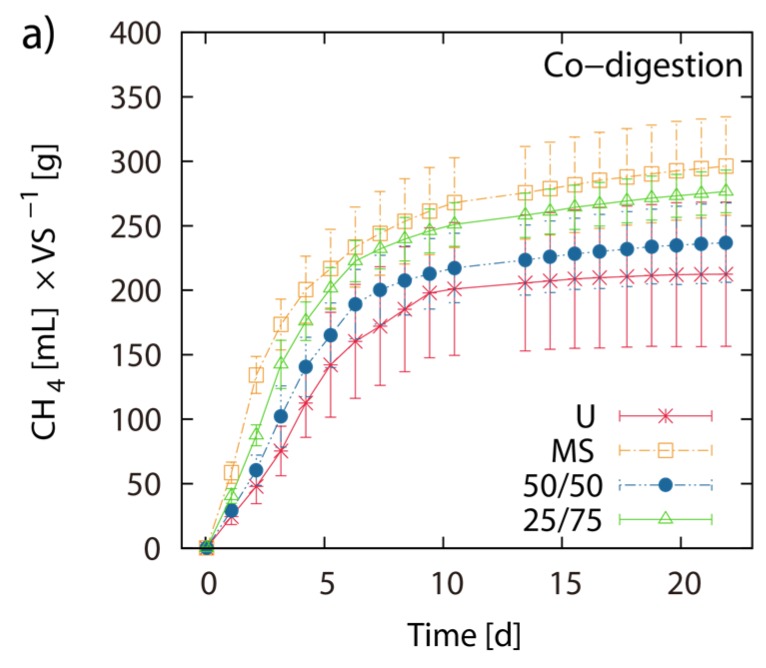

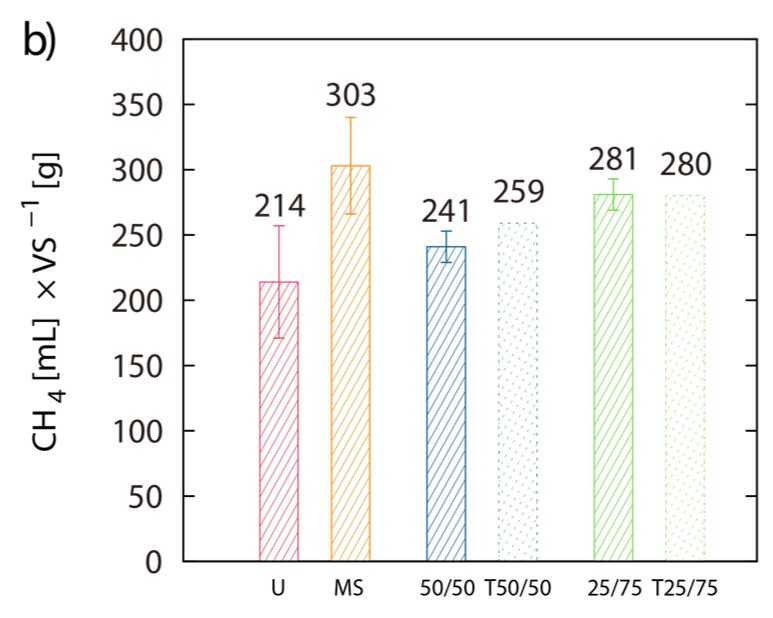
Co-digestion with maize silage: Net accumulated methane production of digestion from LJW (LJ-U), maize silage (MS-U) and their respective blends, 50% LJ/50% MS (50/50) and 25% LJ/75% MS (25/75) (**a**); (**b**) shows the histogram of the real final net methane yield in mL·g^−1^ VS algae biomass, along with the theoretical BMPs (T). The values of the final methane yield are reported in Table 3 and were taken at Day 22.

Single digestion of MS and U-3 yielded 303 mL and 214 mL CH_4_ g^−1^ VS respectively. The 50/50 and the 75/25 blends of MS/LJW showed CH_4_ yields of 241 mL (T = 259 mL) and 281 mL (T = 280 mL) g^−1^ VS respectively (Figure 4a,b). The measured BMP results are placed beside their theoretical counterparts (T(50/50) and T(75/25)), which are based on the calculation of the respective single digestion results (Figure 4b). Comparing theoretical and obtained BMPs from the MS/LJW blends, no improvement in biomethane recovery was noted.

### 2.4. Evaluation of Methane Production Dynamics

The rate of methane formation was determined for the respective batch experiments using the formulas stated in Section 4.8.3. Three reference milestones were set in the batch process denoting the time needed until 50%, 70% and 90% of the maximum BMP (taken after Day 22) was generated (T_50_, T_70_, T_90_). These time points were compared to each other and are displayed in Table 3. Table 3 also displays the total final BMP of the batch experiments including standard deviation. When comparing the different value sets, the dynamics appear similar to each other. No great differences in process dynamics were noted between the different approaches for biomethane production over time.

**Table 3:** Kinetic decay constants and related values of degradation dynamics (time until 50%, 70% and 90% of maximum BMP (T_50_, T_70_, T_90_) were produced) for all batch experiments. Maximum BMP is stated with standard deviation (SD). An increase or decrease of BMP for pre-treated LJW as compared with BMP of untreated LJW are indicated in %.

Experiment	Substrate	K (day^−1^)	T_50_	T_70_	T_90_	BMP [mL·g^−1^]	SD	+/−
E1	U-1	0.2568	2.7	4.7	9.0	172	20	-
E2	U-2	0.403	1.7	3.0	5.7	178	25	+3% ^†^
E3	U-3	0.1792	3.9	6.7	12.8	214	43	+24% ^†^
E1	LJW-0.05 M-20 °C	0.2929	2.4	4.1	7.9	171	22	±0% ^†^
E1	LJW-0.05 M-50 °C	0.2787	2.5	4.3	8.3	180	7	+5% ^†^
E1	LJW-0.05 M-80 °C	0.2476	2.4	4.2	8.0	149	26	−13% ^†^
E1	LJW-0.1 M-20 °C	0.2836	2.4	4.2	8.1	156	12	−9% ^†^
E1	LJW-0.1 M-50 °C	0.2713	2.6	4.4	8.5	176	10	+2% ^†^
E1	LJW-0.1 M-80 °C	0.2571	2.7	4.7	8.0	163	23	−5% ^†^
E1	LJW-0.5 M-20 °C	0.2165	3.2	5.6	10.6	170	22	±0% ^†^
E1	LJW-0.5 M-50 °C	0.2198	3.1	5.5	10.5	150	10	−13% ^†^
E1	LJW-0.5 M-80 °C	0.1712	4.0	7.0	13.4	168	17	−2% ^†^
E2	LJW-HCl-80 °C-pH 1.2	0.29	2.4	4.1	7.9	169	15	−2% ^†^
E2	LJW-FGC-80 °C-pH 1.2	0.2621	2.6	4.6	8.8	168	20	−2% ^†^
E2	LJW-0.2 M-100 °C	0.2643	2.6	4.6	8.7	180	5	+5% ^†^
E3	MS-untreated	0.2643	2.6	4.6	8.7	303	37	-
E3	MS/LJW-50/50	0.2047	3.4	5.9	11.2	241	12	−7% ^‡^
E3	MS/LJW-75/25	0.2297	3.0	5.2	10.0	281	12	±0% ^‡^

^†^ Deviation from U-1 (in %); ^‡^ Deviation from theoretical BMP based on single BMP results (in %).

### 2.5. Continuous Fermentation Studies—Laboratory-Scale

**Figure 5:**
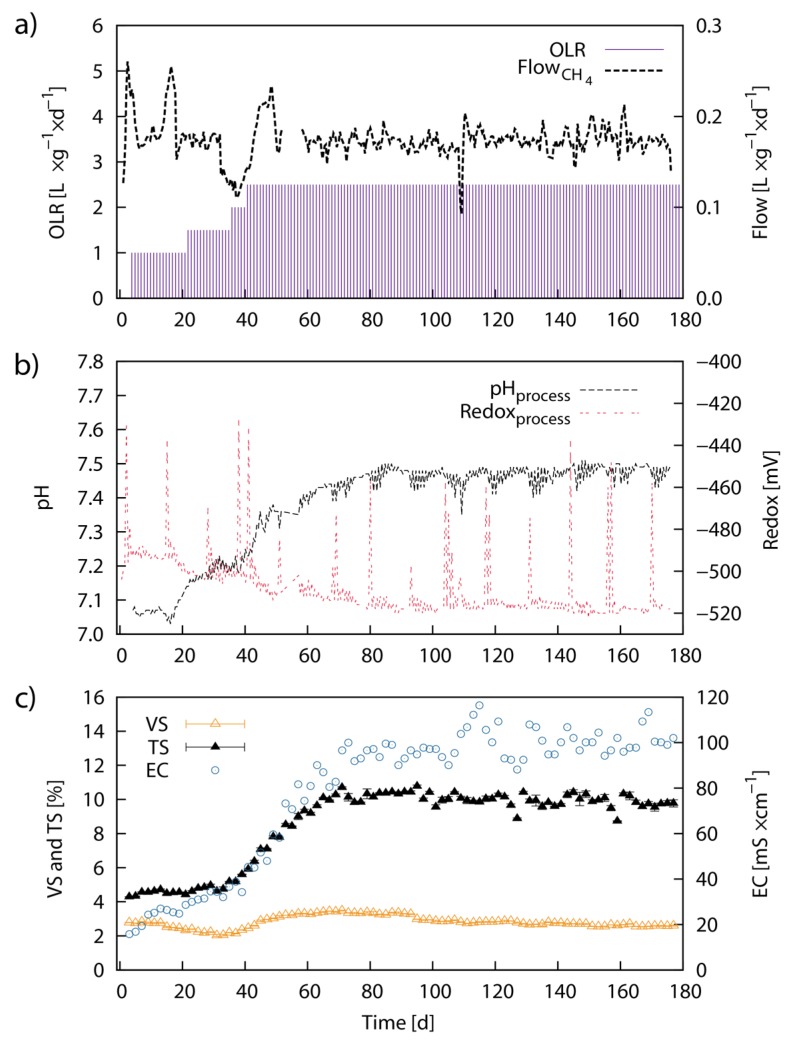
Continuous anaerobic digestion of *Laminaria japonica* waste in laboratory-scale: Laboratory-scale continuous AD of untreated LJW showing the process parameters; cumulative CH_4_ volume (L), specific volumetric CH_4_ production (calculated) per VS feed load (L·g^−1^·day^−1^) (**a**); pH value and redox potential in mV (**b**); conductivity EC in mS·cm^−1^, VS and TS concentration (% from total weight) (**c**). VS and TS represent average values from three individual measurements and error bars are defined as standard deviation (±SD).

The specific CH_4_ flow rate showed a steady production of around 174 mL·g^−1^·day^−1^ with some initial fluctuations during the first 50 days (Figure 5a). The fluctuations coincided with a stepwise increase of the organic loading rate (OLR). The flow rate then started to level out after a steady OLR was maintained at 2.5 g·L^−1^·day^−1^. Over the 178 days of the experiment, an average CH_4_ of 173 mL·g^−1^ VS (88 mL·g^−1^ TS) was produced. The bioreactor operation was divided into a start-up (P1 to P3) and an experimental phase (P4 to P5). During the start-up phase, the OLR was steadily increased to 2.0 g VS L^−1^·day^−1^. The experimental phase was then used to take the bioreactor towards steady-state conditions. The last phase, P5, completed 2.1-times the HRT. The respective average CH_4_ yields and phase conditions for the different phases of bioreactor operation are listed in Table 4. The value for pH in the bioreactor was initially at pH 7.05 but rapidly rose from Day 18 until around Day 80 to ~pH 7.47. Subsequently, the pH value remained steady (Figure 5a). The redox potential leveled out at −520 mV between Day 1 and Day 80 and remained steady henceforth. The regular pikes in the steady redox potential pattern originated from daily feeding events. Conductivity increased from 15 mS·cm^−1^ to ~100 mS·cm^−1^ between Day 1 and Day 80 and oscillated around this value thereafter until the end of the experiment (Figure 5a). The VS concentration in the bioreactor was initially at 2.8% (w/w), slightly increased to 3.5% and again decreased and steadied out at ~2.6%. TS increased from a steady concentration of 4.5% during the first 30 days to 10.0% between Day 30 and Day 70 and remained steady at this mark henceforth. The initial ratio of VS/TS was 65% but changed during the course of the experiment to a final ratio of 26.6% (Figure 5a).

**Table 4:** Operating conditions of the lab-scale and pilot-scale bioreactors with the respective specific CH_4_ production rates, organic loading rates and hydraulic retention times during the different phases of operation.

Mode	Phase	Time (Days)	CH_4_ Production (mL·g^−1^·day^−1^ VS)	OLR (g·L^−1^·day^−1^)	HRT (d)	Times HRT
Lab-scale	P1	3–22	191	1.0	62.5	0.3
	P2	23–35	165	1.5	62.5	0.2
	P3	36–40	124	2.0	62.5	<0.1
	P4	41–89	178	2.5	62.5	0.8
	P5	90–175	173	2.5	40	2.1
Pilot-scale	P0	~150	Pre-run	-	-	-
	P1	4–50	189	2.0	32	1.4

**Figure 6:**
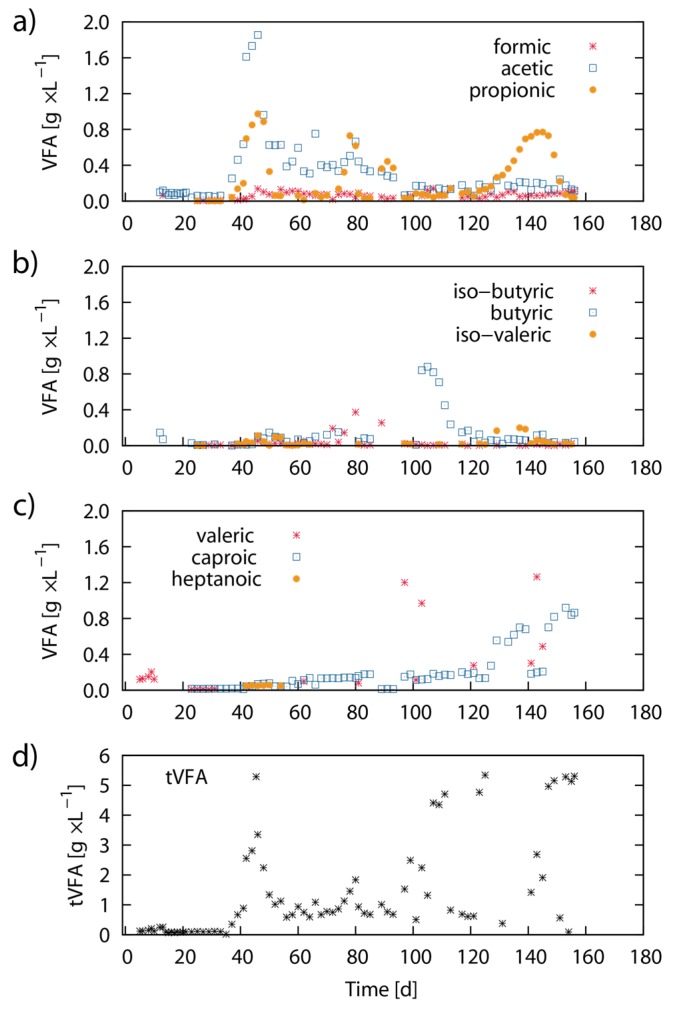
Dynamics of VFA concentration: The graph shows the distribution of VFA concentration (g·L^−1^) for formic acid, acetic acid, propionic acid (**a**); butyric acid, iso-butyric acid, iso-valeric acid (**b**); valeric acid, caproic acid and heptanoic acid (**c**); The last subfigure shows the total sum concentration of VFAs (**d**).

Figure 6 shows results from the respective volatile fatty acid (VFA) concentrations in the fermenter sludge during the experimental period. During the first 35 days of the experiment, all measured VFA concentrations were low. Acetic acid and propionic acid concentrations increased thereafter and showed peaks of 1.9 g·L^−1^ (Day 45) and 1.0 g·L^−1^ (Day 45) respectively. Subsequently, the elevated acetic acid and propionic acid concentrations slowly decreased until Day 100. Propionic acid showed a second concentration peak between Day 125 and Day 155 (Figure 5a). Butyric acid and caproic acid were found in elevated concentrations in the bioreactor between Day 90 to Day 115, and Day 125 to Day 155, respectively. The peak concentrations for butyric acid and caproic acid were 0.9 g·L^−1^ (Day 105) and 0.9 g·L^−1^ (Day 153) respectively (Figure 5b,c). After Day 156, no further data for VFA concentration was available.

### 2.6. Analysis of Fermentation Residue

Table 5 shows the elementary analysis results of LJW fermentation residue (LJWR). The sludge sample was taken at Day 178 (last day of the experiment) as bioreactor content from the laboratory-scale continuous trial. The fermentation residue of LJW contained around 10% TS, with around 2% organic matter, resulting in a low ratio of 20% VS/TS compared with common digestate [52]. The total concentration of carbon (C) was 13% (from TS) and total nitrogen (N) concentration was 3% (from TS). Generally, C and N concentrations were lower in LJWR than those in common digestate. Other macronutrient concentrations diverged from the reference data with potassium (K) and sulfur (S) showing higher concentrations (2.5-fold and 2-fold, respectively), while phosphate (P) (~0.1-fold), Mg (equal) and Ca (~0.3-fold) were present in equal or much lower concentrations. Micronutrients were in the negligible range, with the exception of Fe which was 33-times higher than the declaration limit for fertilizer compounds according to DüMV. The concentration of heavy metals showed values which were below the limitation references.

**Table 5:** Macro- and micronutrient composition and heavy metal concentration of LJW fermentation residue (LJWR), average energy crop digestate (digestate) [52] and minimum and limiting concentration for component declaration according to DüMV (DüMV).

Category	Element	LJWR	Digestate	DüMV	Unit
BioAbfV	Lead (Pb)	5	2.9	150	mg·kg^−1^ TS
	Cadmium (Cd)	1	0.26	1.5	
	Chromium (Cr)	17	9.0	300	
	Copper (Cu)	11	69	70	
	Nickel (Ni)	5	7.5	80	
	Mercury (Hg)	<0.04	0.03	1	
	Zinc (Zn)	46	316	500	
Macronutrients	Phosphorous (P)	2180	25,700	300	
	Potassium (K)	238,000	71,400	500	
	Magnesium (Mg)	11,000	12,000	300	
	Calcium (Ca)	13,300	30,000	500	
	Sulfur (S)	10,300	4710	300	
	C/N ratio	4.3:1	6.4:1		
	Total carbon	13	43		% TS
	Total nitrogen	30,000	67,140	1000	mg·kg^−1^ TS
Micronutrients	Molybdenum (Mo)	1.8		2	mg·kg^−1^ TS
	Iron (Fe)	3330		100	
	Cobalt (Co)	2.0		4	
	Selenium (Se)	0.2			
	Manganese (Mn)	170		200	

### 2.7. Continuous Fermentation Studies—Pilot-Scale

**Figure 7:**
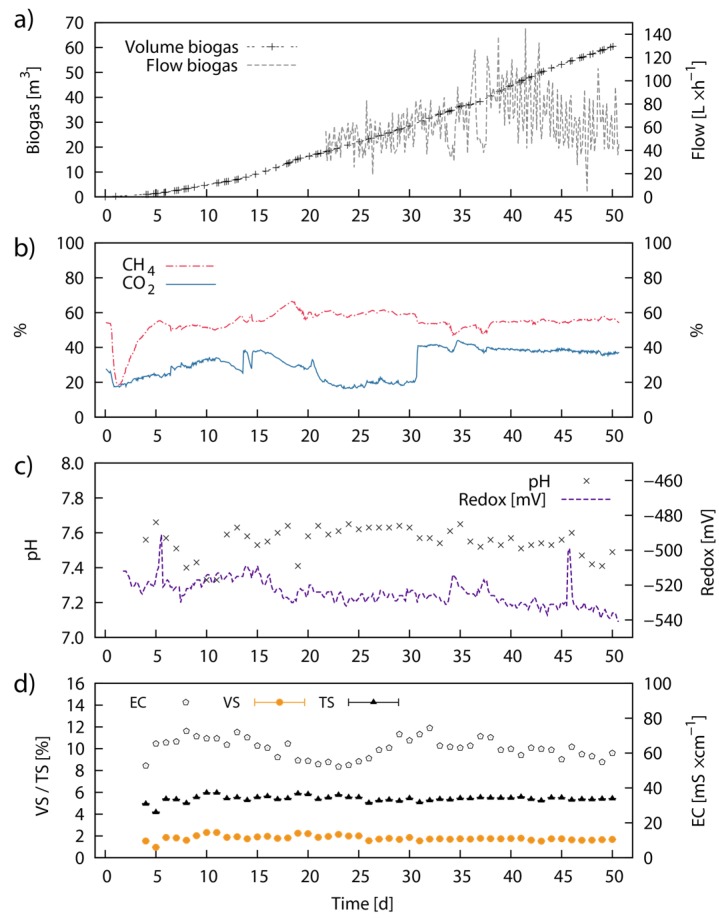
Continuous anaerobic digestion of Laminaria japonica waste in pilot-scale: Pilot-scale continuous AD of untreated LJW over time showing the process parameters; cumulative biogas volume (m^3^), volumetric biogas flow rate (L·h^−1^) (**a**) volumetric CH_4_ and CO_2_ concentration (%) (**b**); pH value and redox potential in mV (**c**); VS, TS concentration (% from total weight) and conductivity (mS·cm^−1^) (**d**). VS and TS represent average values from three individual measurements and error bars are defined by standard deviation (±SD).

The biogas flow rate recordings were started at Day 22 and showed an increase in flow rate from 45 to 85 L·h^−1^ until Day 43. Subsequently, the flow rate decreased again and steadied at ~60 L·h^−1^ until the end of the experiment. The total average biogas flow rate was 63 L·h^−1^, corresponding to 1.21 m^3^ biogas day^−1^ (Figure 7a). Over the 50 days of the experiment, around 60.46 m^3^ of biogas were produced and a total of 165 kg VS (418 kg TS) of LJW was used for feeding, providing an average biogas yield of 370.3 L·kg^−1^ VS (146.3 L·kg^−1^ TS). The CH_4_ and CO_2_ ratio in the biogas leveled out at 55% (average was 54%) and 38% respectively from Day 31 onwards (Figure 7b). The specific CH_4_ yield over the 50 days of the experiment summed up to 189 L·kg^−1^ VS (75 L·kg^−1^ TS). The detailed operating conditions are displayed in Table 6. The values for pH in the bioreactor oscillated between pH 7.4 and pH 7.6, with an average of pH 7.55. The redox potential was around −530 mV for most of the time and slightly declined to −540 mV at the end of the experiment (Figure 7c). The conductivity remained around 62 mS·cm^−1^ throughout the experiment with small divergences between 55 and 70 mS·cm^−1^ (Figure 7d). The VS and TS concentrations in the bioreactor were constant around 1.8% and 5.4% (w/w) respectively setting the VS/TS ratio to 33% (Figure 7d).

**Table 6:** List of pretreatment conditions applied in this work for acid hydrolysis of LJW.

Name	Reaction Time	Temperature	Medium	Concentration	pH after PT
U-1, U-2, U-3	-	-	H_2_O	-	-
PT-20	2 h	20 °C	HCl	0.05 M	4.0
PT-50	2 h	50 °C	HCl	0.05 M	4.3
PT-80	2 h	80 °C	HCl	0.05 M	4.5
PT-20	2 h	20 °C	HCl	0.1 M	2.9
PT-50	2 h	50 °C	HCl	0.1 M	2.9
PT-80	2 h	80 °C	HCl	0.1 M	3.1
PT-20	2 h	20 °C	HCl	0.5 M	0.5
PT-50	2 h	50 °C	HCl	0.5 M	0.6
PT-80	2 h	80 °C	HCl	0.5 M	0.7
HCl-100	2 h	100 °C	HCl	0.2 M	1.2
HCl-80	2 h	80 °C	HCl	pH 1.2	1.2
FGC	2 h	80 °C	FGC	pH 1.2	1.9

## 3. Discussion

### 3.1. LJW Biomass Composition and Theoretical BMP

The elementary composition of LJW showed that the biomass was generally suitable to AD. The higher concentration of mineral substances might even be conducive to AD as they provide an improved supply of nutrients to the microorganisms. However, the increased sulfur concentration potentially endangers the anaerobic digestion process due to the formation of cell-toxic H_2_S [19]. The C/N ratio of 10.5:1 was lower than the optimal range that is favorable for AD, as stated in the literature [20] and indicates a relative surplus of nitrogen in the biomass. The high concentrations of molybdenum, manganese and iron were expected to have a favorable effect on AD due to their importance as trace elements for microbial degradation performance [53]. Composition analysis showed that LJW consisted mainly of carbohydrate (39.2%), with shares of 11.4% protein and 0.3% lipids. Total organic matter was 50.9% and inorganic matter accounted for 49.1%. The share of fermentable organics was similar compared to the whole algae samples used in the AD experiments reported in the literature, with organic fractions between 43% and 69% (dry weight) [14,23,54]. The share of carbohydrates was comparable to the usual values found in brown seaweed [14]. The theoretical methane potential deducted from the composition analysis summed up to 391 mL g^−1^ VS (199 mL·g^−1^ TS). The heavy metal concentrations of LJW were similar to that of maize, except for chromium which displayed a considerably higher concentration.

### 3.2. Effect of Acid Hydrolysis on BMP

The effective BMP from AD of LJW was between 149 mL and 214 mL·g^−1^ VS, where the highest results were obtained for biomethanation of untreated LJW biomass. The pretreatment strategies applied to LJW did not show a groundbreaking effect on yield. Some pretreatment approaches even impaired biomethane conversion, reducing final methane recovery by up to −13%. Impaired performance, or the lack of improved performance, of BMP through pretreatment of LJW might be connected to the presence of higher Na^+^ and Cl^−^ concentration, originating from the pretreatment (HCl) and pH neutralization step (NaOH). The use of FGC instead of industry-grade HCl for pretreatment showed no difference in final BMP. However, since no improvement effects were observed for any of the thermo-acidic pretreatment approaches, this result is in agreement with the other findings. When compared to the calculated maximum theoretical potential, the efficiency of methane recovery was between 38% and 55%. Considering the waste-type origin of the biomaterial and the fact that a biomethane conversion efficiency of ~73% (85% at its maximum) is considered an adequate value for maize silage [55], the results obtained from LJW look acceptable. During the industrial processing of LJW valuable organics (algae leaves) might have been lost or removed which lowered the biomethane conversion efficiency during AD. Considering that LJW contained many stipes and roots, parts of the macroalgae which don’t host many ready fermentable compounds, this may further explain the diminished biomethane recovery rates. Data on CH_4_ recovery from *Laminaria japonica* or other members of the *Laminariales* genera are frequent in the literature and concentrate on laboratory-scale trials, mostly in batch, and some in small continuous systems. Chynoweth *et al*. [56] proposed 260–280 mL and 390–410 mL BMP g^−1^ VS for mesophilic AD of *Laminaria* spp. and *Macrocystis* spp. respectively. Nielsen *et al*. [33] produced 340 mL CH_4_ g^−1^ VS from *Saccharina latissima* under thermophilic process conditions. Diverging from the observations in this study, acid hydrolysis pretreatment applied on *L. japonica* had an effect on the saccharification rate and improved microbial biohydrogen and bioethanol production [57,58]. Hydrothermal depolymerization of alginate (contained in brown seaweeds) was performed by Aida *et al*., (2010) [59] but required reaction temperatures of 180–240 °C. These temperature conditions were neither available nor applied in our study. The lack of BMP improvement with the use of pretreated LJW may be explained by foregoing treatment of LJW during the industrial processing of algae, which reduced the readily fermentable compounds in LJW and left the biomass in an already disintegrated condition. With regards to biomethanation, the loss of fermentable compounds and the partial fractionation of LJW made a pretreatment step in this case ineffective. This is underlined by the observation of improved biomethane recovery from untreated LJW over different batch experiments in this work (+3% and +24%), which suggest an adaptation of the inoculum microorganisms to the algal substrate. Microbial adaptation to surrounding conditions is a commonly known effect in microbial anaerobic digestion and is often reported in the biogas literature [27,35].

### 3.3. Effect of Co-Digestion with Maize Silage on BMP

The co-digestion of LJW with MS did not show any change regarding CH_4_ recovery. Comparing the theoretical calculated values and the experimentally measured BMP, the co-digestion blends yielded more or less equal quantities of biomethane as the single digests of maize silage and LJW. A secondary, or derivative, co-digestion effect might be observed in continuous AD where long-term effects become visible. Batch trials do not allow the testing of substrate deficiency effects or component shortage (e.g., trace elements) as these occur only during long-term operation [51]. Bioreactors exclusively supplied with maize silage are known to show signs of trace element shortage in the course of operation, in particular for molybdenum [53]. In the case of LJW, the macroalgal biomass contains a three-fold higher molybdenum concentration compared to that of maize and could present a potentially interesting co-substrate. Other studies in the literature have tested co-digestion approaches in batch trials which have shown more significant effects on the quality of biomethane recovery. The co-digestion of *Laminaria digitata* with cattle manure improved the specific methane yield considerably [60], while Taihu blue algae co-digested with corn straw yielded +62% [61]. Furthermore, the co-digestion of *Ulva sp.* with waste activated sludge resulted in +26% more biomethane [23].

### 3.4. Effect of Acid Hydrolysis and Co-Digestion on Methane Formation Dynamics

A changing pattern in CH_4_ formation dynamics could not be observed when applying acid hydrolysis to LJW. The velocities of methane formation from untreated and pretreated biomaterial were in similar range. Co-digestion of LJW/MS showed a dynamics pattern that reflected the average from the two individual MS and LJW patterns. A general observation was the complete lack of adjustment phase (Δ lag phase) after the substrate feeding, which was recognizable in all respective approaches. Methane formation started shortly after substrate addition without delay. The microbial decay of untreated LJW biomass worked well and pretreatment or co-digestion effects did not accelerate methane formation. The reason for the good degradation performance of untreated biomaterial might lay in the preceding industrial treatment during the processing of the seaweed, as stated in Section 4.2.

### 3.5. Biomethane Production from LJW in Continuous Anaerobic Digestion (Laboratory-Scale)

The long-time use of untreated LJW as a substrate in continuous anaerobic digestion showed that fermentation of LJW was possible under mesophilic process conditions covering 2.1 hydraulic retention times, which comes close to the 3 hydraulic retention times needed to show reactor stability [35]. However, the bioreactor proved to be stable during the 178 days of the experiment operation exclusively on LJW biomass. The average specific CH_4_ yields obtained were between 124 mL·g^−1^ VS and 191 mL·g^−1^ VS, depending on the phase of operation, with an overall average of 173 mL·g^−1^ VS. These findings coincided with the results obtained from the batch trials. The measured process parameters indicated biochemical process stability, underlining the hypothesis of smooth bioreactor operation. The values for conductivity increased over time and were situated in a higher range (70–100 mS·cm^−1^) than the standard ones [62]. However, this fact did not seem to affect bioreactor functionality. The increase in conductivity might be related to an increased dissolution of salt compounds during the stepwise approximation to steady-state. The salt compounds were likely to originate from the LJW biomass, which consists of higher potassium concentrations as detected during elementary analysis of LJW. The concentration dynamics of VFA showed smaller concentration peaks during the experiment which indicate minor or local reactor disturbances. However, increasing VFA content was subsequently rapidly degraded and stability conditions were restored. Several works were published addressing the continuous biomethanation of brown algae in laboratory-scale, however none were found to use *Laminaria japonica* as substrate material. Research on *L. japonica* was performed with the aim for use in fermentative hydrogen or bioethanol production [57,58]. Hinks *et al*. [35] tested the continuous AD of brown seaweed, *Laminaria hyperborea*, and yielded 140–230 mL CH_4_ g^−1^ VS after completing three hydraulic retention times. Hanssen *et al*. [63] conducted continuous experiments on *L. hyperborea*, *Laminaria saccharina* and *Ascophyllum nodosum* and yielded 280 mL, 230 mL and 110 mL CH_4_ g^−1^ VS respectively. Both Hanssen *et al*. [63] and Hinks *et al*. [35] operated their bioreactors at shorter HRTs (20–25 days) compared to this study (40–62 days) and also applied reduced organic loading to the bioreactors (OLR between 1.0 and 1.75 g·L^−1^·day^−1^). Edyvean *et al*. [40] digested seaweed waste from alginate extraction in a two-stage fermenter system and yielded 195 mL and 198 mL of CH_4_ g^−1^ VS for the mesophilic primary and the thermophilic secondary biodigester respectively. Compared to the CH_4_ recovery in the works of Hinks *et al.* [35], Hansson *et al.* [63] and Edyvean *et al.* [40], the biomethane production from LJW was within the same range. The degree of degradation of LJW digestion (~73%) showed an exquisite performance in terms of biomass-to-biomethane conversion, which is comparable with the results obtained from AD of maize silage [55].

### 3.6. Analysis of Fermentation Residue

During a process, the option to further use the fermentation residue is always important to consider, due to its role as either disturbing waste material or valuable biofertilizer. Comparing input (LJW) and output (LJWR) material, the C concentration was reduced by −38%, showing a strong conversion of C in the form of CH_4_ and CO_2_ during process operation. The C reduction rate was equal to that proposed by Menardo *et al*. [64], assessed for different substrate types. The concentration of N was increased by +50%, indicating an accumulation of total N in the bioreactor and shifting the C/N ratio from 10.5:1 to 4.3:1. This observation was the consequence of the N concentration effect whereby C is degraded to CH_4_ and CO_2_ while preserving N [65]. N, P and K are probably the most relevant nutrients for the determination of good fertilizer properties [66,67]. LJWR generally contains high amounts of K but low amounts of N and P. The large share of inorganic compounds in LJWR might have originated from the high inorganic sand and solids content present in the initial LJW biomass. The removal of sand and other disturbing materials would allow further nutrient concentration and an improvement in nutrient ratios. The presence of these materials also decreases the logistical efficiency of fertilizer distribution through the transportation of additional, bioinactive weight. Removal of sand and other process-interfering materials is an important downstream processing step, but can become energy intensive. A promising method has been successfully developed for beached macroalgae harvested along German coastlines which are used for automotive fuel production [68].

To assess more explicitly the quality of LJWR to serve as biofertilizer, the determination of microbial nutrient bioavailability is required. This accounts in particular for NH_4_-N and P compounds, where plants have a preferred form for direct nutrient uptake [69]. The pollutant concentrations of heavy metals were considered uncritical.

### 3.7. Performance in Pilot-Scale Continuous Anaerobic Digestion

The findings gained in the pilot-scale trial confirmed several of the results obtained in laboratory-scale, but also provided some new practical information regarding the upscaling of biomethanation from LJW. The pilot-scale experiment showed a CH_4_ recovery of 189 L·kg^−1^ VS, equaling the range of 173 mL·g^−1^ VS obtained during laboratory-scale bioreactor operation. The ratios of CO_2_ and CH_4_ in the generated biogas were 38% and 55% respectively and thus in agreement with the values for carbohydrate-rich substrates and macroalgae stated in the literature [29,42]. The only comparable pilot-scale study on continuous AD of *Laminaria* spp. was performed by Matsui *et al*. (2006) [42] who used beached macroalgae to run a pilot biogas plant with a daily input of 0.2–1.0 tons of fresh biomass. The hydraulic retention time in the Matsui *et al*. [42] work was between 15 and 25 days and thus shorter than in this study. However, the results of the present work, such as biogas composition and CH_4_ recovery, were by all means comparable to the results obtained by Matsui *et al*. (2006) [42]. The CH_4_ production rate in the Matsui *et al*. [42] study summed up to 22 m^3^·t^−1^ of fresh macroalgae, with a biogas composition of 60% CH_4_ and 40% CO_2_ [29]. Anticipating a VS content of ~10% in fresh *Laminaria* spp., as noted by Matsui (2010) [70] in a succeeding publication, the CH_4_ production rate calculates to 220 m^3^·t^−1^ VS. Methane recovery of LJW, as a dispensable and waste-type material, reached almost 86% of the value obtained by Matsui *et al*. (2006) [42].

The considerable amount of sand and insoluble solids in the digester sludge probes suggested an accumulation in the biodigester. Similar observations were reported by Matsui *et al*. [42] when operating a large-scale pilot plant with beached *Laminaria* spp. and *Ulva* spp. This fact, *per se*, does not pose a problem to the AD process itself since these materials are not involved in microbial activity and the biochemical reactions. However, with accumulation of solids in the biodigester, the active reaction volume is reduced and in consequence, the CH_4_ production rate per volume is lowered. Further, the presence of sand impairs the quality of fermentation residue and contributes to accelerated wear-out of technical and mechanical plant devices. A coarse removal of sand and other inorganic solids preceding AD is therefore recommended [68]. This point was also stressed by Wei *et al*. [14] when using macroalgae for biofuel production or phytochemical extraction. During our pilot study, the clogging of pipes and pumps was observed several times. These originated through plastic braids contained in the LJW biomass, cross-linking to a plug-like structure. The problem was resolved by catching the impurities in rotating stirrers, where immobilization of plastic braids was achieved through furling and accumulation. The interfering substances were then properly removed to avoid further technical issues. Generally, such problems can be circumvented by choosing suitable high-definition devices (e.g., chaffing pumps) or through further upscaling of the system to operational plant devices, which would change the relative size of particles. Small-scale, mobilized, container-shaped plug-flow bioreactors, combined with a dry fermentation technique that is less affected by coarse ingestate materials, might present a suitable way to manage the bulky composition of LJW. Additionally, with regards to substrate availability and substrate transportation logistics, a small-scale unit organization might be more suitable.

A co-digestion of terrestrial energy crops with a 25% seaweed share (by weight), of similar biomethane potential as LJW, could decrease land-use for bioenergy in Germany by approximately 290.000 hectares for energy crops and approximately 211.000 hectares for maize. This represents 10%–14% of the cultivated land where renewable resources are generated for energy use [4].

## 4. Experimental Section

### 4.1. Macroalgae Biomass and Maize Silage

**Figure 8:**
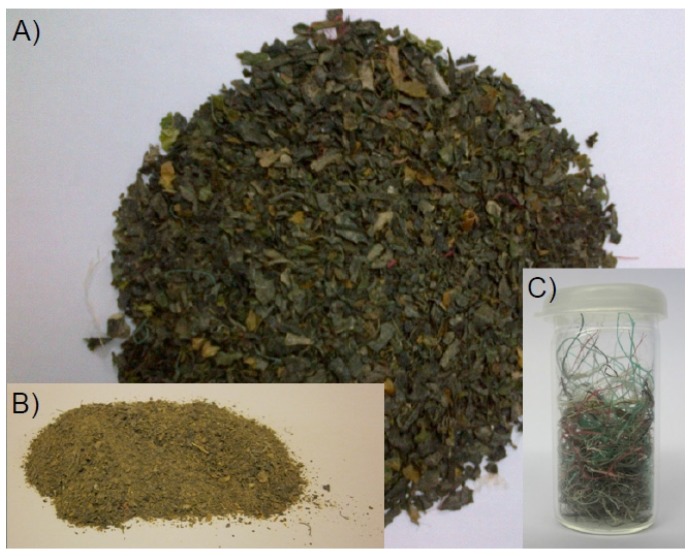
Laminaria japonica waste substrate: Dry and native *Laminaria japonica* waste material as obtained from Qingdao CoDo International Limited, Qingdao, China (**a**) containing plastic braids (**c**). For laboratory use, the biomaterial was shredded and sieved to a particle size of <0.5 cm (**b**).

The *Laminaria japonica* waste (LJW) biomaterial was obtained from Phytolutions GmbH and originated from Qingdao CoDo International Limited, Qingdao, China. It was classed as “unprocessed substandard, sun dried (water content ≥ 22%) and shredded biomass”. Chopped by blades it contained besides whole algae particles also stipes and roots as well as impurity levels of sand, polyester-kelp culture rope fragments from holdfasts (Figure 8c). The dewatered biomass was composed of small *Laminaria japonica* flakes (Figure 8a) with a particle size of <2 cm. Prior to laboratory-scale application, the biomass was milled in a Grindomix GM200 (Retsch, Haan, Germany) and sieved to a particle size of <0.5 cm (Figure 8b). Some inorganic impurities, such as sand particles and small stones, were removed in this step to facilitate technical handling and to avoid damages to the experimental setup. The VS content of processed LJW was 50.9% (from total solids (TS)). For the pilot-scale experiment, the LJW biomass was used in its native state as obtained from Phytolutions GmbH showing a VS content of 39.5% (from TS).

Maize silage was obtained from a local biogas plant operator in Osterholz-Scharmbeck/Lower-Saxony, Germany. It consisted of coarsely shredded whole-plant particles (approximately 0.5–2 cm particle size). After sampling, the biomass was divided into small shares and stored at −20 °C until further use.

### 4.2. Inoculum Sludge

The inoculum sludge for the batch experiments (inoculum-1) was obtained from a mixture of digester sludge from a waste water treatment plant in Farge/Bremen, Germany and from a biogas plant in Osterholz-Scharmbeck/Lower Saxony, Germany. The inoculum was mixed with digester sludge adapted to the macroalgal substrate used in previous batch experiments in a ratio of approximately 3:1.

The inoculum used for the continuous laboratory-scale trial (inoculum-2) originated from the above described sources of digester sludge and was blended to a starting concentration of 2.9% VS and 4.3% TS. Fibers and coarse material were removed from the inoculum prior to experimentation to avoid technical problems during bioreactor operation.

The inoculum used for the continuous pilot-scale experiment (inoculum-3) underwent a similar blending procedure as inoculum-1 and -2. Inoculum-3 was pre-adapted to the LJW biomass over a period of 5 months, where 1.5 kg VS LJW per m^3^ active volume was added to the bioreactor system every other day, aiming to keep VS and TS values (data not shown) steady in the system at ~1.5% VS and ~5% TS. Inoculum-1 to -3 originated from the same sources of seeding sludge, but the respective blends for the different experiments were prepared individually since the experiments were not conducted simultaneously. Therefore, the inocula were similar but not identical in composition.

### 4.3. Flue Gas Condensate

The flue gas condensate was obtained from the middle-calorific power plant in Bremen, Germany (swb AG) using paper, wood, synthetics and residual package material as combustible. The FGC was a waterish and clear liquid with a pH value of 0.92. A precise content analysis of the FGC was not performed, but based on the information of the source material (exhaust gases from combustion), FGC is supposed to mainly contain inorganic acids, such as sulfuric/sulfurous acid and nitric/nitrous acid. The FGC was stored at room temperature until further use.

### 4.4. Acid Hydrolysis Pretreatment

After mechanical treatment as described in Section 4.1 the biomass was chemically and thermally pretreated. To apply acid hydrolysis, the powdery material was blended with the acidic solution and thoroughly mixed and heated using a thermo-controlled laboratory shaker for stirring (HT Aquatron, Infors AG, Bottmingen, Switzerland, 175 rpm). Pretreatment reaction time was 2 h for all approaches. After cooling, the pH of the solution was adjusted with NaOH to a value between 6.8 and 7.1 if necessary. The prepared substrate suspensions were stored at 4 °C until use in the experiments. The entire macroalgae-liquid suspension was poured into the digester during the feeding procedure. An overview of the respective types of pretreatment applied in this work is listed in Table 6.

### 4.5. BMP Tests and Batch Array

The batch array was composed of major units each consisting of: Digester (1), CO_2_ removal (2), silica gel unit (3) and gas volume sensor (gasUino) (4). NaOH (3 M) was used to scrub CO_2_ and H_2_S from the native biogas. Thymolphtalein indicator (0.4%) was added to NaOH to check the CO_2_ absorption performance. The volumetric CH_4_ recordings were conducted using a gasUino apparatus and corrected to standard conditions (0 °C and 1013.15 hPa) [22]. The gasUino gas counter works on a liquid displacement principle where 75% NaCl solution (pH 1) was used as a gas blocking solution [71]. To ensure gas-tight conditions, butyl rubber tubing was used for the connections.

The BMP trials were conducted at a mesophilic temperature range (37 °C ± 1 °C). All experiments were carried out in triplicates (2000 mL bottles), including positive (microcrystalline cellulose (mcc)) and negative (inoculum alone) controls. Fermenter bottles were filled with 1600 mL of seeding sludge and were pre-incubated for one week to reduce remaining methane potential. At feeding, 400 mL of pre-treated algae-media suspension or untreated algae-water suspension was added to fill up to a total volume of 2000 mL. Respecting the VDI 4630 guideline [51], the final VS inoculum concentration was set between 1.5% and 2% (w/w). Accordingly, the quantity of added substrate was set to VS_substrate_ = VS_inoculum_/2 [51]. Digester tanks were flushed with N_2_ for 2 min after the feeding procedure to establish anaerobic conditions. Methane production was recorded for at least 22 days. The requirement for a correct validation of the data sets was a 75%–80% minimum efficiency of biomethane recovery for the positive control [51].

### 4.6. Continuous Setup CSTR 10 L

A 10 L Biostat MD fermenter (B. Braun, Melsungen AG, Melsungen, Germany) was used to perform continuous laboratory experiments. The setup consisted of a continuously stirred tank reactor (CSTR) with a heating jacket to maintain a steady process temperature (mesophilic at 37 °C ± 1 °C). The stirring speed was set to 100 rpm. The gas outlet was connected to a subsequent CO_2_/H_2_S scrubbing unit (NaOH), a gas drying unit and finally to a gasUino gas volume sensor (identical order as in the BMP trials). The volumetric CH_4_ recordings were performed with a gasUino apparatus and corrected to standard conditions (0 °C and 1013.15 hPa). Butyl-rubber tubes were used for the connections. A pH and a redox process electrode were permanently inserted into the digester tank for online data measurement. The substrate feeding and sludge sampling were managed through an in- and outlet pipe. Sludge sampling and feeding were performed manually, once a day (every 24 h ± 3 h), using a technique preventing biogas leakage or loss of head space pressure in the system.

### 4.7. Pilot-Scale Bioreactor System

A 1700 L fermenter (own construction, Ocean Lab, Jacobs University Bremen, Bremen, Germany) was used to perform continuous pilot-scale experiments. The setup was located outdoors and consisted of a fully stirred bioreactor with a heating- and insulation system, to maintain steady process temperature (mesophilic at 37 °C ± 1 °C), and 800 L substrate blending tank and a post-fermentation tank to hold the digestate. The gas outlet was connected to a H_2_S absorbing unit (activated charcoal) and two IR-based gas analyzers were used to measure the CH_4_ (BCP-CH4, BlueSens gas sensor GmbH, Herten, Germany) and CO_2_ (BCP-CO2, BlueSens gas sensor GmbH) concentrations. The volumetric biogas recordings were measured using a drum gas counter (TG05/2, Ritter GmbH, Hildesheim, Germany) and corrected to standard conditions (0 °C and 1013.15 hPa). PVC tubes were used for the gas connections. Process parameters (pH, redox potential) were recorded using in-process electrodes (Jumo GmbH, Fulda, Germany). A process control unit (own construction, Jacobs University Bremen) linked to the *Sensus* software (Michael Hofbauer Elektronische Systeme) was used for data recording and automatized digester feeding. Continuous feeding was performed every 4 h using a peristaltic pump (SPX-32, Watson-Marlow/Bredel, Rommerskirchen, Germany). Sludge samples were taken for analysis manually once per day (every 24 h ± 3 h) to measure conductivity, VS and TS of the digester content.

### 4.8. Analytical Methods and Calculations

#### 4.8.1. Measurement of Volatile Solids and Total Solids

Concentrations of TS and VS were determined by drying and calcinating samples at 105 °C and 550 °C respectively, for 24 h (P300, Nabertherm, Lilienthal, Germany). Single data points are an average value of three individual sample measurement (*n* = 3). Error bars and deviation (±) were calculated with standard deviation from triplicate values.

#### 4.8.2. Data Treatment from Methane Production

The methane volume and the cumulative methane production for the batch experiments were calculated by subtracting the average blank sample values accordingly. The volume was adjusted to standard conditions using the normalization formula used by Barbot *et al*. [22]. For batch experiments, all given data points consist of an average of three individual values (triplicates, *n =* 3). Error bars and deviation (±) were calculated with standard deviation of triplicate values. The biomethane production for the continuous experiments was calculated from the daily biomethane volume (24 h) produced and related to the daily feed quantity of LJW.

#### 4.8.3. Calculations for Comparison of Methane Formation Dynamics in Batch

A combination of first and second order kinetics was used to investigate the dynamics of methane formation for the respective approaches. Equation (1) was used to determine the decay constants *k* and to calculate the respective points in time (days) when 50%, 70% and 90% of the total BMP was generated. The modified Gompertz Formula (2) was used to determine the lag phase Δ for each experimental approach [72].

(1) $$G\left(t\right)=G_{0}\;\times (1-e^{-kt})$$

(2) $$G\left(t\right)=G_{0}\;\times \left[-e\left[\left(\frac{u}{G_{0}}\right)\times \left(\Delta -t\right)+1\right]\right]$$

where *G(t)* is the cumulative CH_4_ yield at time *t* (mL CH_4_ g^−1^ VS), *G_0_* is the total BMP of the substrate (mL CH_4_ g^−1^ VS), *k* is the CH_4_ production decay rate constant (day^−1^), *t* is the time of BMP test in days and Δ is the lag phase (days).

## 5. Conclusions

Residual *Laminaria japonica* waste (LJW) biomaterial, originating from an industrial process, was tested as ingestate biomass for microbial anaerobic conversion to biomethane. Laboratory trials in batch and continuous mode showed a biomethane recovery of 174 mL·g^−1^ VS from untreated LJW. This valorization pathway seems acceptable considering that LJW is categorized as a waste-type biomaterial and contains a diminished amount of readily fermentable compounds compared to whole-plant *Laminaria* biomass. The application of thermo-acidic pretreatment prior to anaerobic digestion failed to improve either the final CH_4_ yield or the dynamics of CH_4_ production. Similar findings were observed when testing co-digestion of LJW with maize silage, which did not increase the respective BMPs. The fermentation residue from digested LJW shows an attractive potential to serve as biofertilizer, being free of heavy metal contaminants and rich in potassium, sulfur and iron. A pilot-scale study revealed some important information on the practicability of upscaling AD of LJW. With a biomethane recovery of 189 L·kg^−1^ VS and a biogas composition of 55% CH_4_ and 38% CO_2_, the findings were in accordance with our own laboratory-scale trials. We proved that upscaling of LJW biomethanation is possible relying solely on the source material as a unique substrate in its “native” state, with identical recovery of benefits as seen in the laboratory-scale applications. Difficulties in the pilot-scale process operation were encountered more on the technical rather than on the biochemical aspect of the trial. Insoluble coarse materials led to jamming of technical plant devices and the bioreactor sand content lowered the biomethane conversion efficiency per active biodigester volume.
